# Toxical Effects of Copper and Zinc

**Published:** 1845-06

**Authors:** 


*Toxical Effects of Copper and Zinc.*—M. Blandet states that the copper colic, although rare in hospitals, is very common in workshops, and that all apprentices are attacked with it. It appears to be produced by the inhalation of pulverized copper. It is generally an apyretic affection, characterized by colics, with extreme prostration, and sometimes by diarrhœa, sometimes by constipation. Its ordinary duration is forty-eight hours. In the Paris workshops milk is used as a preservative. The treatment consists in emollients, or slight purgatives, according to the symptoms. In copper foundries, on the afternoon of the fusing day, or on the morning following, the workmen employed experience muscular pains, cephalalgia, general lassitude, vomiting, and rigors, which end in copious sweats and slight febrile reaction. These symptoms, M. Blandet attributes to the zinc, which is fused along with the copper to form bronze, &c. The very high temperature which is resorted to, in order to fuse the combined metals, gives rise to the formation of vapours of zinc, which become oxidized and fill the workshop. In the fusion of zinc alone these accidents are not observed, it not being necessary to raise the temperature so high.

